# Expression of estrogen-related receptors in ovarian cancer and impact on survival

**DOI:** 10.1007/s00432-021-03673-9

**Published:** 2021-06-05

**Authors:** Susanne Schüler-Toprak, Florian Weber, Maciej Skrzypczak, Olaf Ortmann, Oliver Treeck

**Affiliations:** 1 Department of Gynecology and Obstetrics, University Medical Center Regensburg, Landshuter Str. 65, 93053 Regensburg, Germany; 2 Department of Pathology, University Medical Center Regensburg, Franz-Josef Strauß Allee11, 93053 Regensburg, Germany; 3 Second Department of Gynecology, Medical University of Lublin, Jaczewskiego 8, 20-090 Lublin, Poland

**Keywords:** Estrogen-related receptors, Ovarian cancer, Overall survival, Progression-free survival, Tissue microarray

## Abstract

**Purpose:**

This study further approaches the role of estrogen-related receptors (ERRs) in ovarian cancer. Protein expression of ERRα, ERRβ and ERRγ in ovarian cancer was assessed and was correlated with ovarian cancer markers, steroid hormone receptors and cancer-associated genes. Additionally, we examined to what extent expression of ERRs affects survival of ovarian cancer patients.

**Methods:**

For this purpose, we established a tissue microarray from 208 ovarian cancer patients and performed immunohistochemical analyses of the mentioned proteins.

**Results:**

ERRα and ERRγ protein could be detected at different levels in more than 90% of all ovarian cancer tissues, whereas expression of ERRβ was observed in 82.2% of the cases. ERRα was found to positively correlate with ovarian cancer marker CEA (*p* < 0.005) and ERRγ correlated with ERα (*p* < 0.001). Univariate survival analyses revealed that ERRα expression did not affect overall (OS) or progression-free survival (PFS) of ovarian cancer patients. In contrast, higher expression of ERRβ in serous ovarian cancers was found to lead to a significantly decreased OS (*p* < 0.05). The strongest impact on survival was exhibited by ERRγ. Lower expression of this receptor in women with serous ovarian cancers indicated significantly increased OS compared to those with higher levels of ERRγ (*p* < 0.05). Multivariate survival analyses revealed ERRγ as an independent prognostic marker regarding OS of patients with serous ovarian cancer.

**Conclusion:**

Our data demonstrating that ERR proteins are frequently expressed in ovarian cancer and high levels of ERRβ and ERRγ significantly decreased OS of serous ovarian cancer patients suggest that these proteins might be interesting therapy targets in this cancer entity.

**Supplementary Information:**

The online version contains supplementary material available at 10.1007/s00432-021-03673-9.

## Introduction

Ovarian cancer is the leading cause of death from a gynaecological malignancy in the developed world (Siegel et al. 2018). Due to missing screening methods and the aggressive behavior of the disease, the majority is diagnosed in advanced stages (Torre et al. 2018). Ovarian cancer has been shown to be influenced by steroid hormones. Estrogens activate growth in ovarian cancer cells via ERα which is often overexpressed in this cancer entity (Chan et al. 2017; O’Donnell et al. 2005). In contrast, both expression and specific activation of ERβ which is downregulated in most ovarian cancer cases, reduces ovarian cancer cell proliferation (Halon et al. 2011; Schüler-Toprak et al. 2017; Treeck et al. 2007).

To date, knowledge on the function and expression of estrogen-related receptors (ERRs) α, β and γ in ovarian cancer is sparse. Generally, ERRs are transcription regulators. They use estrogen response elements (EREs) and extended ERE half-sites termed ERR response elements (ERREs) (Ariazi and Jordan 2006). However, endogenous estrogens are no ligands of these orphan receptors (Ariazi and Jordan 2006). ERRs interact with ERα and several other nuclear receptors (Tanida et al. 2015; Yamamoto et al. 2012). Thereby, among others, a vast number of different genes modulating metabolic processes are regulated and several different pathways are controlled (Ranhotra 2012).

ERRα which has attracted the greatest attention to date, acts as a master regulator of cellular metabolism, thereby also promoting tumor growth (Liu et al. 2018). ERRα modulates estrogen responsiveness and substitutes for ER activities in breast cancer and was found to be critical for growth of ERα-negative breast cancer cells (Kraus et al. 2002; Liu et al. 2018; Stein and McDonnell 2006). It increases breast cancer cell migration, proliferation, and tumor development, activates estrogen-responsive genes in endocrine-resistant tumors and associates with unfavorable biomarkers in breast cancer, suggesting ERRα as novel target for therapy of breast cancer (Ariazi et al. 2002; May 2014). With regard to ovarian cancer, a very limited number of studies exist, reporting that targeted inhibition of ERRα hindered epithelial-to-mesenchymal transition and stem cell properties of ovarian cancer cells (Lam et al. 2014). A small study including 33 ovarian cancer patients suggested a reduced overall survival (OS) of patients with high ERRα levels (Sun et al. 2005). In an mRNA-based study, the levels of ERRα transcripts increased with clinical stages in ovarian cancers (Fujimoto et al. 2007).

ERRβ suppresses growth of prostate cancer cells via p21(WAF1) induction making it a potential therapeutic target in prostate cancer (Yu et al. 2008). In breast cancer, ERRβ is upregulated by estrogens in an ERα-dependent manner and inversely correlated with OS of breast cancer patients (Madhu Krishna et al. 2018). With regard to ovarian cancer, few data have been published on this receptor. A study on the mRNA-level stated that ERRβ levels in ovarian cancer tissue were too low to determine reliably (Fujimoto et al. 2007). Thus, further studies on ERRβ in ovarian cancer are necessary.

ERRγ binds Bisphenol A (Tohmé et al. 2014) and affects estrogen responsiveness in endometrial cancer cells (Yamamoto et al. 2012). It correlates with favorable markers in breast cancer (Ariazi et al. 2002). With regard to ovarian cancer, again only limited studies exist. The small study mentioned above including 33 patients suggested that high expression of this receptor is associated with a longer progression-free survival (PFS) (Sun et al. 2005).

Given that there are only few data on the significance of ERR protein levels in ovarian cancer, in this study, we examined protein expression of ERRα, β and γ in 208 ovarian cancer samples, performed correlation analyses with ovarian cancer markers, steroid hormone receptors and other cancer-associated genes and finally performed Kaplan–Meier analyses to elucidate the effect of their expression levels on survival of ovarian cancer patients.

## Materials and methods

### Tissue samples

We included ovarian cancer samples collected in the Department of Pathology of the University of Regensburg. Generally, Caucasian women with sporadic ovarian cancer and available information on grading, stage, and histological subtype from 1995 to 2013 were included. Patients’ clinical data were available from tumor registry database information provided by the Tumor Center Regensburg (Bavaria, Germany). This high-quality population-based regional cancer registry was founded in 1991 and covers a population of more than 2.2 million people of Upper Palatinate and Lower Bavaria. Information about diagnosis, course of disease, therapies, and long-term follow-up are documented. Patient data originate from the University Hospital Regensburg, 53 regional hospitals, and more than 1000 practicing doctors in the region. Based on medical reports, pathology, and follow-up records, these population-based data are routinely being documented and fed into the cancer registry. The retrospective study was approved by the institutional review board “Ethics Committee University of Regensburg”.

### Tissue microarray and immunohistochemistry

The tissue microarray (TMA) was created using standard procedures that have been previously described (Mirlacher and Simon 2010). From all patients included in this study, an experienced pathologist evaluated H&E sections of tumor tissue and representative areas were marked. From these areas, core biopsies on the corresponding paraffin blocks were removed and transferred into the grid of a recipient block according to a predesigned array of about 60 specimens in each of five TMA paraffin blocks.

**Fig. 1:**
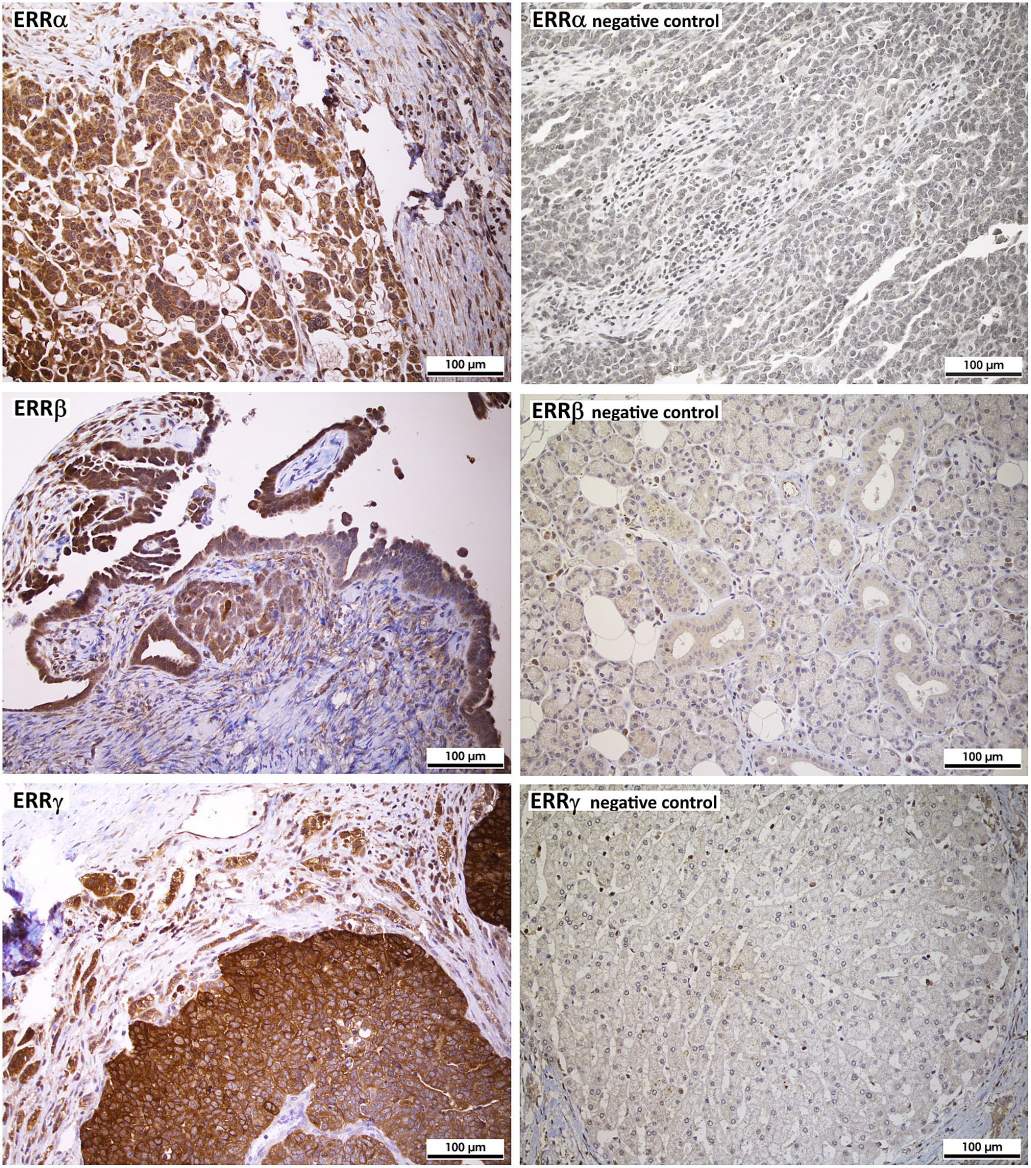
Analysis of protein expression of the indicated ERRs in ovarian cancer tissue microarrays (TMAs) by immunohistochemistry (200 × magnification). On the left side, three representative examples of positive staining of the indicated receptors in serous high-grade ovarian cancer tissue (G3) is shown. On the right side, examples for negative staining are shown (top: negative ovarian cancer tissue, middle: salivary gland, bottom: liver). The absence of ERRβ and γ expression in these tissues is confirmed by data of www.proteinatlas.org

For immunohistochemistry, 4 μm sections of the TMA blocks were incubated with the indicated antibodies according to the mentioned protocols in the given dilutions (Supplemental table S1), followed by incubation with an HRP-conjugated secondary antibody and another incubation with 3,3′-diaminobenzidine (DAB) as substrate, which resulted in a brown-colored precipitate at the antigen site. An experienced clinical pathologist evaluated immunohistochemical staining according to localization and specificity (Fig. 1). For determination of staining intensity of ERRα and ERRγ, a score from 0 (negative) to 3 (strongly positive) was used. Since staining intensities for ERRβ were generally lower, a score from 0 to 2 was used. For steroid hormone receptors ERα, nuclear ERβ and PR, the immunoreactivity score according to Remmele et al. was used (Remmele and Stegner 1987). Expression of proliferation marker Ki-67 using antibody clone MIB-1 was assessed in the percentage of tumor cells with positive nuclear staining. Her2/neu expression was scored according to the DAKO score routinely used for breast cancer cases. EGFR was scored according to Spaulding et al. on a 4-tiered scale from 0 to 3 (Spaulding and Spaulding 2002). For p53 and polyclonal CEA, the “quickscore” was used, where results are scored by multiplying the percentage of positive cells (*P*) by the intensity (*I*) according to the formula: *Q* = *P* × *I*; maximum = 300 (Charafe-Jauffret et al. 2004). CA-125 and ERβ were described as positive or negative, irrespective of staining intensity.

### Statistical analysis

Apart from multivariate survival analyses, statistical analysis was performed using GraphPad Prism 5^®^ (GraphPad Software, Inc., La Jolla, CA, USA). The non-parametric Kruskal–Wallis rank-sum test was used for testing differences in receptor expression among three or more groups. For pairwise comparison the non-parametric Mann–Whitney U rank-sum test was used. Correlation analysis was performed using the Spearman correlation coefficient. Univariate survival analyses were performed using the Kaplan–Meier method. The chi-squared statistic of the log-rank was used to investigate differences between survival curves. Hazard ratios were calculated using the Mantel–Haenszel method. P values below 0.05 were considered statistically significant. Multivariate Cox regression survival analysis was performed using IBM^®^ SPSS^®^ Statistics 25 (SPSS^®^, IBM^®^ Corp., Armonk, NY, USA) using the Enter method.

## Results

### Characteristics of included patients and their tumors

Tissues from 208 Caucasian women with sporadic ovarian cancer were used in this study. Median age at diagnosis was 63.5 years (29–91 years). Table 1 shows the histopathological characteristics. Serous ovarian cancers represent 64.9% of the tumors and 58.8% were grade 3. Most of the cancers were diagnosed in FIGO (International Federation of Gynecologists and Obstetricians) stages III and IV (31.25% and 24.04%, respectively). During the median follow-up of 1180 days, 80 relapses and 62 deaths were documented. While median relapse-free survival was 1044 days, median overall survival (OS) was 1079 days.

**Table 1:** Stages and histopathological characteristics of the included ovarian cancer cases

Characteristics	Number of patients	(%)
Ovarian cancer patients	208	
FIGO stage		
FIGO I	22	10.58
FIGO II	8	3.85
FIGO III	65	31.25
FIGO IV	50	24.04
Unknown	63	30.29
Histological subtype		
Serous	135	64.90
Mucinous	6	2.88
Endometrioid	10	4.81
Clear cell	3	1.44
Undifferentiated	54	25.96
Histological grade		
G2	53	25.48
G3	122	58.65
Unknown	33	15.87

### Comparison of ERR expression in ovarian cancer and normal ovary using publicly available mRNA data

**Fig. 2:**
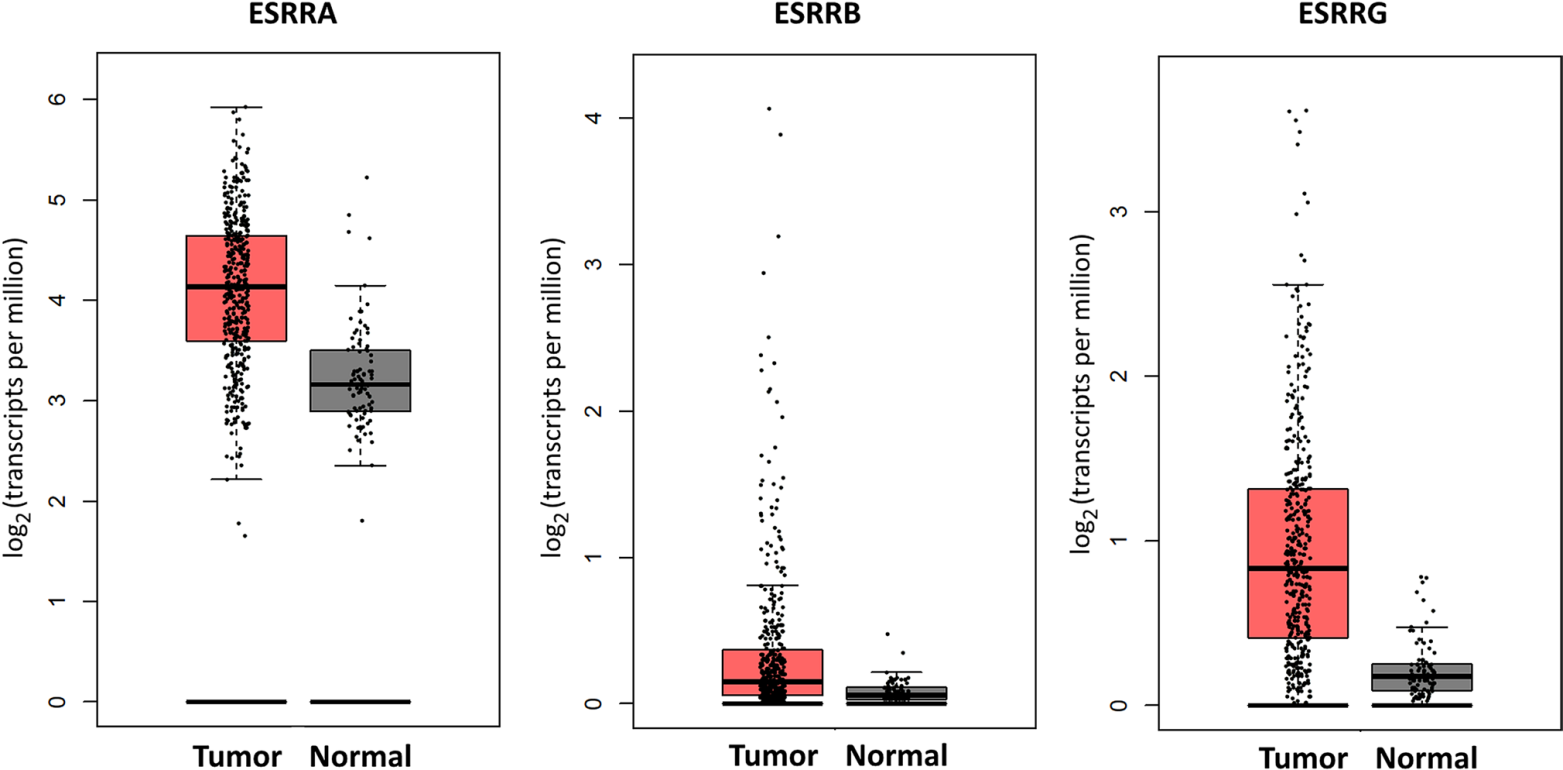
Comparison of ERR mRNA levels in ovarian cancer and normal ovarian tissue based on open source TCGA and GTEx mRNA data using the GEPIA online tool (http://gepia.cancer-pku.cn). Indicated are the ERR genes ESRRA, coding for ERRα protein and ESRRB and ESRRG, coding for the receptors ERRβ and ERRγ, respectively. Compared is the expression in 426 ovarian cancer tissues and 88 normal ovarian tissues

Given that we could not collect a sufficient amount of normal ovarian tissues, we decided to use the benefits of publicly available gene expression data and were thereby able to compare ERR mRNA expression in 426 ovarian cancer tissues and 88 normal ovarian tissues. This analysis of open source TCGA and GTEx mRNA data using the GEPIA online tool (http://gepia.cancer-pku.cn) revealed a notable overexpression of ERRα, β and γ mRNA in ovarian cancer tissue (Fig. 2).

### Expression of estrogen-related receptors in ovarian cancer tissue

**Fig. 3:**
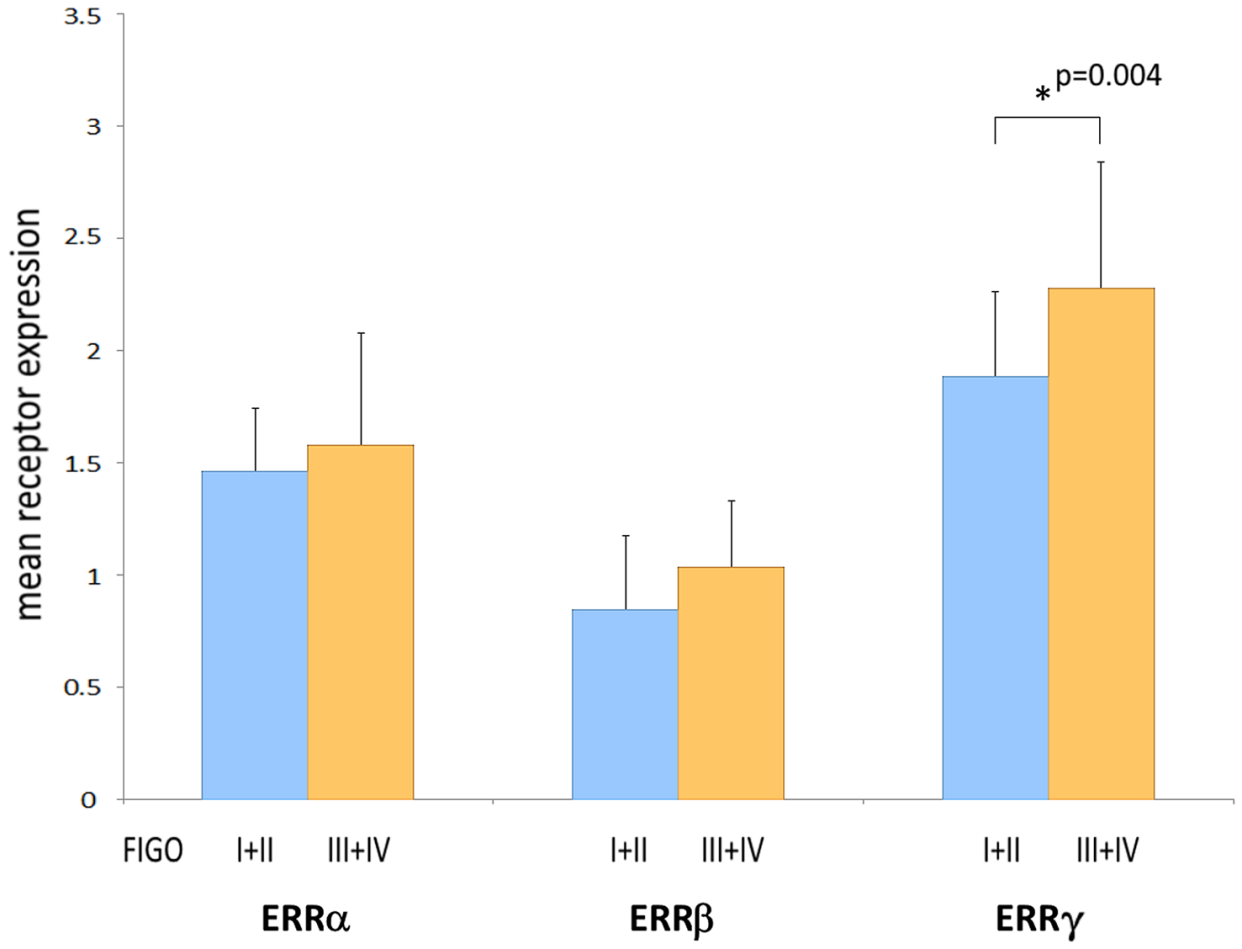
ERR protein expression levels in different FIGO groups as assessed after IHC detection of the indicated proteins on tissue microarrays (TMAs) with 208 ovarian cancer samples. Shown is the mean value of expression scores

We demonstrate ERRs to be widely expressed in most ovarian cancer tissues as assessed on the protein level by means of immunohistochemistry of tissue microarrays (TMAs). Positive staining of ERRα was found in 91.8% of all cases (39.4% weak staining, 45.7% moderate and 6.7% strong staining). ERRβ was detected in 82.2% of all tumors, among them 68.8% with clearly positive but weaker staining, the further samples exhibited stronger staining for this receptor. ERRγ was expressed in 96.6% of all ovarian cancer samples (10.1% with weaker staining, 50.9% with moderate and 35.6% with strong staining (Table 2a). Only considering the largest subgroup of serous ovarian cancer, we detected similar frequencies. Thus, overall staining intensities were highest for ERRγ, followed by ERRα and lowest for ERRβ both in all ovarian cancer samples and in the serous subgroup. With regard to ERRγ, we observed a statistically significant higher mean staining intensity (2.28) in tumors with FIGO stages III or IV than in those staged I or II (1.88) (*p* = 0.004) (Fig. 3). Including serous ovarian cancers only, we also observed an increased mean staining intensity of ERRγ in FIGO stages III and IV (2.27) than in FIGO staged I or II tumors (1.93) (*p* = 0.47) (Table 2b). In contrast, we did not observe any significant difference in expression levels of any ERR between G2 and G3 graded tumors or with patients with different nodal status. Moreover, invasion of lymph vessels or the venous system did not depend on expression of any ERR. ERR expression in metastases also did not significantly differ from their levels in primary tumors (data not shown).

**Table 2:** Expression of estrogen-related receptors (ERRs) in ovarian cancer

a) Rate of expression of the indicated receptors. Shown are the numbers of positive samples in relation to the total numbers of ovarian cancer cases (*n* = 208) or of the subgroups analyzed and the corresponding percent value (in brackets)					
			ERRα	ERRβ	ERRγ
All		All	191/208 (91.8%)	171/208 (82.2%)	201/208 (96.6%)
		G2	52/53 (98.1%)	35/53 (66.0%)	52/53 (98.1%)
		G3	114/122 (93.4%)	115/122 (94.2%)	119/122 (97.5%)
		FIGO I + II	30/30 (100%)	21/30 (70.0%)	27/30 (90.0%)
		FIGO III + IV	107/115 (93.0%)	103/115 (89.6%)	115/115 (100%)
Serous		Serous	128/135 (94.8%)	117/135 (86.7%)	132/135 (97.8%)
		G2	23/23 (100%)	16/23 (69.6%)	23/23 (100%)
		G3	90/97 (92.7%)	86/97 (88.7%)	95/97 (97.9%)
		FIGO I + II	16/16 (100%)	13/16 (81.2%)	16/16 (100%)
		FIGO III + IV	77/84 (91.7%)	72/84 (85.7%)	82/84 (97.6%)

(b) Mean receptor expression levels in all ovarian cancer specimens and in the serous subgroup. A non-parametric Kruskal–Wallis rank-sum test was used for testing differences in receptor expression of the indicated ERRs among the groups. *P* values < 0.05 were considered statistically significant (indicated using bold font)							
		ERRα		ERRβ		ERRγ	
		Mean	*p* value	Mean	*p* value	Mean	*p* value
All	G2	1.552	0.699	0.923	0.428	2.230	0.987
	G3	1.611		1.056		2.314	
	FIGO I + II	1.461	0.438	0.846	0.101	1.884	**0.004**
	FIGO III + IV	1.580		1.035		2.277	
Serous	G2	1.606	0.776	0.909	0.549	2.347	0.284
	G3	1.562		0.987		2.183	
	FIGO I + II	1.466	0.648	0.866	0.487	1.933	**0.047**
	FIGO III + IV	1.558		0.971		2.271

### Correlation of ERR expression with steroid hormone receptors, ovarian cancer markers and other cancer-related genes

To further analyse the role of ERRs in ovarian cancer, we examined correlations between the expression levels of ERRα, β and γ with levels of ERα, ERβ, PR, CA125, CEA, CA72-4, EGFR, HER2, Ki-67 and p53 in all ovarian cancer tissues. By means of Spearman’s rank correlation analysis, we observed a positive association between all ERRs. ERRα correlated with ERRβ (rho = 0.4785, *p* < 0.0001) and with ERRγ (rho = 0.3504, *p* < 0.0001), whereas ERRβ was also associated with expression of ERRγ (rho = 0.4317, *p* < 0.0001) (Table 3). Furthermore, we observed a positive association of ERRα with cancer marker CEA (rho = 0.254, *p* < 0.005). ERRγ was associated with expression of ERα (rho = 0.2858, *p* < 0.001). Expression of the other proteins mentioned above was not significantly associated with any ERR.

**Table 3:** Association of ERRα, β and γ with the indicated steroid hormone receptors and cancer-associated genes in ovarian cancer tissues assessed by means of Spearman’s rank correlation analysis

	ERRα	ERRβ	ERRγ
ERRα		rho = 0.4785 95% CI 0.3457–0.5926 *p* < 0.0001	rho = 0.3504 95% CI 0.203–0.4823 *p* < 0.0001
ERRβ	rho = 0.4785 95% CI: 0.3457–0.5926 *p* < 0.0001		rho = 0.4317 95% CI 0.2944–0.5515 *p* < 0.0001
ERRγ	rho = 0.3504 95% CI 0.203–0.4823 *p* < 0.0001	rho = 0.4317 95% CI 0.2944–0.5515 *p* < 0.0001	
ERα	n.s	n.s	rho = 0.2858 95% CI 0.1346–0.424 *p* < 0.001
CEA	rho = 0.254 95% CI 0.09935–0.3967 *p* < 0.005	n.s	n.s

Shown are the rank correlation coefficient (rho), the 95% confidence interval (CI) and the *p*-value, considered as statistically significant in case of *p* < 0.005 (due to multiple comparison analysis)

Correlations with ERβ, PR, MKI67, TP53, HER2, EGFR, CA-125 and CA72-4 were also tested, but were not statistically significant

### Analysis of ERR expression in ovarian cancer subgroups defined by the level of molecular marker expression

Next, we compared the mean protein levels of ERRα, β and γ in ovarian cancer subgroups with high vs. low expression of the molecular markers examined in this study, like steroid hormone receptors, ovarian cancer markers and proliferation markers. First, we found that mean levels of ERRα and ERRγ were elevated in ovarian cancer specimen with higher ERα expression when compared to the lower expressing subgroup (*p* = 0.02 or *p* = 0.001, respectively) (Table 4). Mean protein levels of ERRα, β and γ were increased in ovarian cancers with higher expression of ERβ (*p* = 0.03, *p* = 0.006 and *p* = 0.003, respectively). Protein levels of ERRα were also elevated in the CEA-high subgroup (*p* = 0.004). In the subgroup with higher expression of CA125, we observed increased ERRγ levels (*p* = 0.011). In ovarian cancers with higher expression of proliferation marker Ki-67, we found a decreased mean ERRα level (*p* = 0.03). ERRγ protein levels were observed to be elevated in tumors with higher expression of tumor suppressor TP53 (*p* = 0.0007). Finally, mean protein expression of ERRα, β and γ was elevated in ovarian cancers with higher expression of HER2 receptor tyrosine kinase (*p* = 0.002, *p* = 0.007 and *p* = 0.013, respectively). No differences in ERR expression levels could be observed between tumor subgroups with different levels of PR, CA72-4 or EGFR.

**Table 4:**
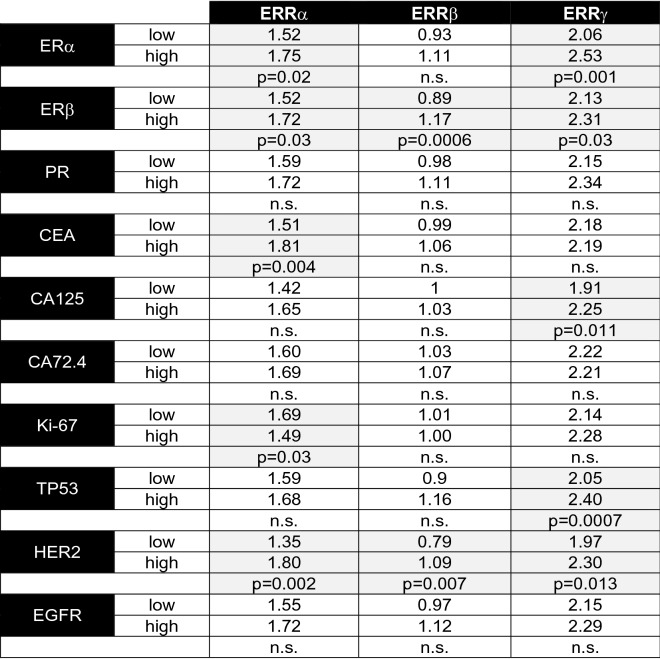
Mean protein levels of ERRα, β and γ in ovarian cancer subject to high and low expression of the indicated genes

Statistical significance was stated in the case of *p* < 0.05 and was highlighted by light gray color

### Survival analyses

#### ERRα expression is not associated with overall or progression-free survival of ovarian cancer patients

When we compared overall survival (OS) of all women with ovarian cancers expressing different levels of ERRα by means of Kaplan–Meier analysis, no significant differences were found (data not shown). We further investigated survival of patients with serous ovarian cancers. However, ERRα expression did not influence OS of these patients in our cohort (data not shown). The levels of this receptor also did not correlate with progression-free survival (PFS), neither when including all ovarian cancer cases, nor when analyzing only serous ovarian cancers.

#### High expression of ERRβ protein in serous ovarian cancer is associated with a significantly decreased overall survival

**Fig. 4:**
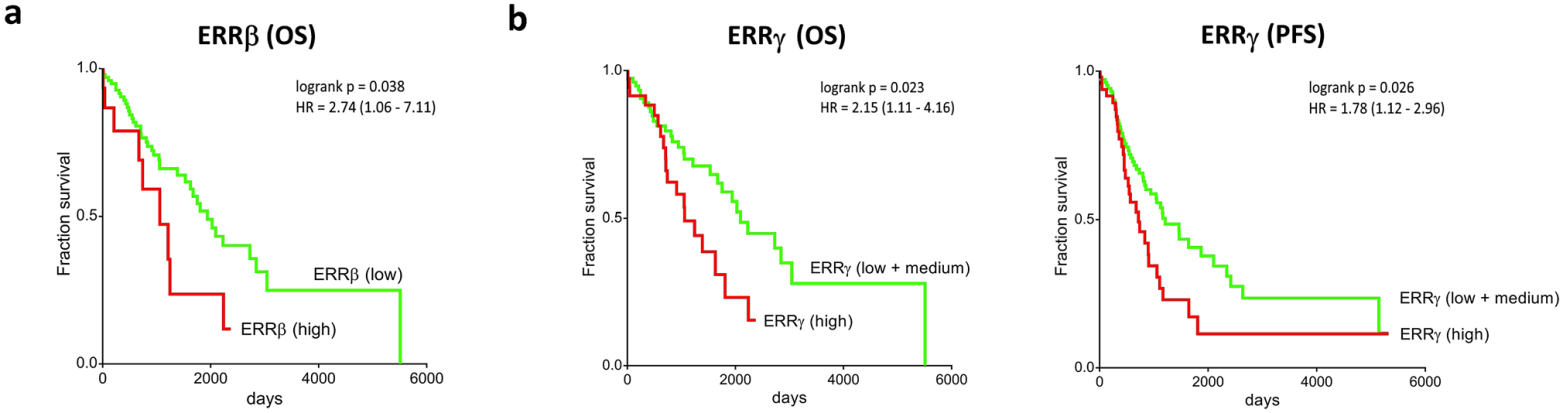
Kaplan–Meier diagrams for survival analyses in relation to ERR protein expression. **a** Overall survival (OS) of patients with serous ovarian cancers with regard to expression to ERRβ. **b** OS and progression-free survival (PFS) of ovarian cancer patients with different tumoral expression of ERRγ; shown is the OS of serous ovarian cancer patients with low and medium expression of ERRγ compared to those expressing high levels of ERRγ and the PFS of all ovarian cancer patients with low and medium expression of ERRγ compared to those expressing high levels of ERRγ. The hazard ratio (HR) is indicated with the 95% confidence interval in brackets

Survival analyses revealed a significantly increased OS of patients with serous ovarian cancers expressing no or low levels of ERRβ compared to those with serous tumors showing higher ERRβ expression (chi-squared statistic of the log-rank, *p* = 0.038). Median survival of patients with serous ovarian cancers expressing high levels of ERRβ was 1058 days, whereas women with tumors with low expression of ERRβ had a median survival of 1938 days (hazard ratio (HR) 2.74; 95% CI 1.06–7.11) (Fig. 4a). However, ERRβ levels did not affect PFS of these patients with serous ovarian cancer (data not shown). We did not observe significant differences in OS or PFS depending on ERRβ expression when analyzing all histologic subtypes (data not shown).

#### ERRγ protein expression levels are associated with OS in patients with serous ovarian cancers as well as with PFS of all patients with ovarian cancer

High expression of ERRγ in women with serous ovarian cancers indicated significantly increased OS compared to those with serous tumors expressing low or medium levels of ERRγ (Chi-square statistic of the log-rank, *p* = 0.023). Median survival of women with high ERRγ expression was 1058 days compared to 2095 days of patients with serous cancers showing lower expression of ERRγ (HR 2.15; 95% CI 1.11–4.16) (Fig. 4b). We did not observe significant differences in OS depending on ERRγ expression when analyzing all samples representing mixed histologic subtypes (data not shown). With regard to PFS, patients differed significantly when comparing all ovarian cancer cases with low and medium ERRγ protein levels to those with high expression of ERRγ (chi-squared statistic of the log-rank, *p* = 0.026). Median PFS of women with ovarian cancers expressing low and medium levels of ERRγ was 1213 days compared to 714 days of women suffering from tumors with high expression of ERRγ (HR 1.78; 95% CI 1.12–2.96) (Fig. 4b). Survival analyses of the subgroup of serous ovarian cancers revealed a trend toward longer PFS for ovarian cancer patients whose tumors showed low expression of ERRγ compared to those with medium and high expression (data not shown).

#### ERRγ is an independent prognostic marker for OS in patients with serous ovarian cancer

Multivariate Cox regression survival analysis was used to further investigate significant results of the beforehand performed univariate analyses. Thus, influence of protein expression levels of ERRβ and ERRγ on OS of patients with serous ovarian cancer was examined. We found a significant effect of ERRγ expression on OS of patients with serous ovarian cancer (HR 1.846; 95% CI 1.097–3.108, *p* = 0.021) making it an independent prognostic marker for OS in this subgroup of patients (Table 5). Protein expression of ERRβ did not have significant effects on OS in multivariate survival analysis (Table 5).

**Table 5:** Multivariate survival analysis of overall survival (OS) of patients with serous ovarian cancer included protein expression of ERRα and ERRβ

	HR	*p* value	95% CI
ERRβ	1.085	0.787	0.601–1.957
ERRγ	**1.846**	**0.021**	**1.097–3.108**

Shown are the results of cox-regression analysis using the enter method (hazard ratio (HR) and 95% confidence interval (CI). Statistical significance is stated in the case of *p* < 0.05 and highlighted using bold font

## Discussion

Increasing evidence supports a clear influence of ERRs on carcinogenesis of different endocrine-regulated tumor entities making these receptors putative therapeutic targets. Until now, little is known about the role of the ERR subtypes ERRα, β and γ in ovarian cancers. We report protein expression of ERRα, β and γ in the vast majority of 208 ovarian cancer samples as assessed by IHC of tissue microarrays (TMAs). Moreover, univariate survival analyses of our data suggest that high levels of ERRβ and ERRγ, but not ERRα, significantly shorten the OS of patients with serous ovarian cancer. ERRγ also negatively affected PFS of all ovarian cancer cases. Multivariate survival analysis points out ERRγ as an independent prognostic marker for OS of patients with serous ovarian cancer.

When investigating the role of ERRs as a possible target in anticancer therapy of ovarian tumors, it is essential to learn more about their expression level and -frequency. Until now, little was known about protein expression of the different ERR subtypes in ovarian cancers. On the protein level, we detected ERRα in 91.8%, ERRβ in 82.2% and ERRγ in 96.6% of all tumors. Previous publications, including a small number of ovarian cancer cases, only investigated ERR expression on the mRNA level. Sun et al. detected mRNA of ERRα in their small study including 33 ovarian cancer cases in 19 of 33 samples (57.6%) (Sun et al. 2005). Only three (9.1%) ovarian cancer samples expressed the ERRβ mRNA. Expression of ERRγ mRNA was observed in 16 of 33 ovarian cancers (48.5%) (Sun et al. 2005). Fujimoto et al. also only found low mRNA expression levels of ERRβ and ERRγ which made further analyses impossible (Fujimoto et al. 2007). When we analyzed open source TCGA and GTEx mRNA data for expression differences of the three ERRs between ovarian cancer and normal ovarian tissue, we found an overexpression of ERRα, β and γ mRNA in ovarian cancer tissue compared to that of normal ovaries. This is in line with data published by Sun et al. (Sun et al. 2005). Heterogeneity of ovarian cancers often limits therapeutic effects. The high frequency of ERR protein expression in ovarian cancer we observed in our study might make these receptors attractive therapy targets.

Among the ERRs, the ERRα subtype attracted the greatest attention to date. In our IHC-based TMA study, ERRα protein expression levels did not affect OS or PFS of the included ovarian cancer patients. In breast, endometrial and ovarian cancer ERRα has been reported to act as a master regulator of cellular metabolism and to thereby also stimulate tumor growth (Liu et al. 2018). Moreover, ERRα was reported to promote cancer migration and metastasis in these endocrine-dependent gynaecological cancers (Liu et al. 2018). In in vitro studies, ERRα was suggested to exhibit pro-metastatic effects in ovarian cancer cells (Wang et al. 2017). In addition, ERRα was reported to activate Snail, a crucial regulator of epithelial–mesenchymal transition (EMT) (Lam et al. 2014). Inhibiting the expression of ERRα in vitro was shown to reduce the migratory capacity of breast, prostate and colon cancer cells, as well as ablation of β-catenin. Thus, the ERRα/β-catenin/WNT11 signaling pathway was suggested to be biologically significant (Dwyer et al. 2010; Zhao et al. 2012). WNT11 has been found upregulated in several cancers and its expression has been previously associated with increased cell migration (Uysal-Onganer et al. 2010). Recent studies demonstrated that WNT11 expression is directly co-regulated with ERRα and β-catenin in several cancer cell lines, which is considered the key mechanism underlying the pro-migratory activity of ERRα (Dwyer et al. 2010; Zhao et al. 2012).

Here, we observed a positive correlation of ERRα protein expression with the ovarian cancer marker CEA. Carcinoembryonic antigen (CEA) is produced during fetal development and functions as a cellular adhesion factor during organ formation (Saeland et al. 2012). It is also involved in cellular adherence and aggregation processes (Abdul-Wahid et al. 2014). CEA acts as a paracrine factor, activating human fibroblasts by signaling through both STAT3- and AKT1-mTORC1 pathways, promoting their transition to the cancer-associated fibroblast phenotype, and enhancing cell migration (Abdul-Wahid et al. 2018; Chen et al. 2019). Moreover, a connection between CEA and the WNT/β-catenin oncogenic pathway has been reported (Chen et al. 2019). Thus, the observed positive correlation between ERRα and CEA expression might support the previously suggested oncogenic features of this receptor, as both trigger migratory and metastatic processes of cancer cells, among others via oncogenic WNT/β-catenin signaling. In line with the previously suggested oncogenic role of ERRα we observed elevated expression levels of ERRα in ovarian cancers with high expression of Ki-67 or HER2 in our cohort. This is also supported by our observation that transcript levels of ERRα are higher in ovarian cancers than in normal ovarian tissue, although ERRα expression did not affect survival in our patients’ cohort.

With regard to ERRβ, high protein expression of this orphan receptor in the subtype of serous ovarian cancers indicated a significantly shorter OS, suggesting an oncogenic effect of ERRβ. Until now, data on the role of this orphan receptor in ovarian cancers, particularly concerning the association of ERRβ expression with survival of ovarian cancer patients, are sparse. In line with our data, a small mRNA-based study observed a tendency toward longer OS and PFS in ERRβ mRNA negative ovarian cancers (Sun et al. 2005).

In line with these observations, suggesting an oncogenic role of ERRβ, we observed an elevated median receptor expression of ERRβ in ovarian cancers expressing high levels of HER2 in our cohort. In contrast to these findings in ovarian cancers, two in vitro studies on breast or prostate cancer cells suggested a tumor-suppressive role of ERRβ (Madhu Krishna et al. 2018; Yu et al. 2008). In breast cancer, a direct interaction between ERα and ERRβ was observed by Tanida et al. (Tanida et al. 2015). Co-expression of ERRβ led to significantly reduced mobility of ligand-activated ERα and significantly repressed ERα-mediated transcriptional activity (Tanida et al. 2015). In that study, ERRβ significantly inhibited E2-stimulated proliferation and expression of bcl-2 in MCF-7 breast cancer cells (Tanida et al. 2015). Moreover, ERRβ was reported to exhibit inhibitory effects on the cell cycle via regulation of p18, p21cip and cyclin D1 in breast cancer cells (Madhu Krishna et al. 2018). In line with these findings, we observed elevated expression of ERRβ in ovarian cancer expressing high levels of the tumor suppressor ERβ.

Thus, these findings and our data suggest carefully balanced functions of ERRβ in ovarian cancer that can be affected by co-regulators. The observed negative effect on survival of ovarian cancer patients with tumors expressing ERRβ suggests that interaction with other influencers might mediate an oncogenic role in vivo. Future studies are strongly required to further elucidate the molecular mechanisms mediating the effect of ERRβ in ovarian cancer.

With regard to ERRγ, we found a highly significant association between high protein expression and shortened OS of patients with serous ovarian cancer in univariate as well as in multivariate survival analysis, suggesting a tumor-promoting role of this receptor in this cancer entity. In contrast to our data suggesting a significant association with shorter PFS, in a small mRNA-based study, it was reported that PFS of women with ERRγ mRNA expressing ovarian cancers was significantly longer than in the ERRγ negative group (Sun et al. 2005). However, the small number of cases in that study and the fact that mRNA levels do not reflect the amount of active protein have to be considered.

Moreover, we observed a significantly higher ERRγ protein mean staining intensity in FIGO stages III and IV than in stages I and II. This is in line with previously published mRNA data, showing higher ERRγ transcript levels in FIGO III and IV ovarian cancers (Sun et al. 2005).

The role of ERRγ in ovarian cancer has not been investigated in detail. In an in vitro study on prostate cancer cells, ERRγ was reported to exhibit anti-proliferative effects. (Yu et al. 2007). In contrast, in line with our data suggesting a tumor-promoting effect of ERRγ protein in ovarian cancer, in a study on breast cancer cells, an oncogenic role of ERRγ has been reported as exogenously transfected ERRγ increased proliferation of MCF-7 cells (Ijichi et al. 2011). In endometrial cancer, estrogen-induced transcriptional activity of the ERE was repressed by ERRγ in ERα-positive cells but was stimulated by ERRγ in ERα-negative cells (Yamamoto et al. 2012). Moreover, a selective ERRγ agonist, DY131, inhibited growth of ERα-positive endometrial cancer cells but promoted that of the ERα-negative cancer cells (Yamamoto et al. 2012), suggesting a subtle harmonized interaction between ERα and ERRγ in the regulation of tumor cell proliferation, which is supported by our observation of a positive correlation between the expression of ERRγ and ERα. Consistent with this, Castet et al. showed that nuclear receptor interacting protein 140 (NRIP140), known to act as co-regulator of ERα and ERRs, differentially regulated ERR activity depending on the target sequence on the promoters (Castet et al. 2006). Regarding the E2-regulation of transcription through ERα/Sp1 interaction, target genes were involved in either positive or negative control of cell proliferation (Castet et al. 2006). Supporting the oncogenic role of ERRγ, we observed elevated expression levels of ERRγ in ovarian cancers expressing high levels of CA125 and HER2.

Taken together, the data showing elevated ERRγ mRNA levels in ovarian cancers, the significantly higher mean staining intensity of ERRγ protein in higher staged tumors compared to those that were detected early as well as the association of high ERRγ protein expression with shorter OS and PFS of ovarian cancer patients suggest a tumor-promoting role of ERRγ in ovarian cancer.

As ERRs have been shown to be promising therapeutic targets in different cancer entities, several specific agonists and antagonists for ERR subtypes have been developed showing convincing effects in vitro (Ariazi and Jordan 2006; Du et al. 2017; Vernier et al. 2020).

## Conclusion

In conclusion, we were able to detect considerable protein expression of ERR α, β and γ in the vast majority of 208 ovarian cancer samples. Moreover, survival analyses showed a significant adverse effect of ERRβ and γ protein expression on OS of serous ovarian cancer patients and pointed ERRγ out to be an independent prognostic marker in this subgroup. This makes these ERRs interesting targets for therapeutic interventions using recently developed pharmacological ERR modulators. Future studies further elucidating mechanisms of action and function of the different ERR subtypes in ovarian cancer as well as in vivo approaches to test the applicability of ERR modulators in treatment of this cancer entity are necessary.

## Supplementary Information

Below is the link to the electronic supplementary material.

Supplementary file1 Table S1: Antibodies used in this study (PDF 16 kb)

## Data Availability

Data available on request from the authors.
