# Supplementary material for: Expression of estrogen-related receptors in ovarian cancer and impact on survival
Source: J Cancer Res Clin Oncol. 2021 Jun 5;147(9):2555–67. doi: 10.1007/s00432-021-03673-9 (PMC8310835; doi:10.1007/s00432-021-03673-9)
Supplement: Supplementary file 1 — Supplementary file1 Table S1: Antibodies used in this study (PDF 16 kb) [file 432_2021_3673_MOESM1_ESM.pdf]

Supplemental Table

Expression of estrogen related receptors in ovarian cancer and impact on survival

Susanne Schüler-Toprak*, Florian Weber*, Maciej Skrzypczak, Olaf Ortmann and Oliver Treeck

Journal of Cancer Research and Clinical Oncology

Corresponding author:

Priv.-Doz. Dr. Susanne Schüler-Toprak

Department of Gynecology and Obstetrics, University Medical Center Regensburg, Landshuter Str. 65, 93053 Regensburg, Germany.

Phone: +49-941-782-3402, fax: +49-941-782-7515,

email: Susanne.Schueler@klinik.uni-regensburg.de; ORCID 0000-0003-4791-2595

Supplemental table 1: Antibodies used in this study

Marker/ Protein	Antibody Clone	Pretreatment	Dilution	Pattern
ERRα	ab93173 (Abcam)	TE 5'	1:50	nuclear
ERRβ	ab19331 (Abcam)	TE 5'	1:50	nuclear
ERRγ	ab49129 (Abcam)	TE 5'	1:50	nuclear
ERα	6F11 (Novocastra)	CC1 64'	1:35	nuclear
ERβ	PPG5/10 (Novus Biologicals)	None	1:20	nuclear/cytoplasmic
CA-125	OC125 (Cell Marque)	CC1 52'	1:1	cytoplasmic/membranous
CEA	A 0115 (Dako)	P1 8'	1:500	cytoplasmic
CA72.4	B72.3 (Alexis Biochemicals)	CC1 36'	1:50	cytoplasmic
EGFR	E30 (Dako)	P1 4'	1:100	membranous
p53	sc-263 (Santa Cruz)	CC1 36'	1:2000	nuclear
Ki-67	MIB-1/M7240 (Dako)	CC1 64'	1:100	nuclear
PR	NCL-L-PGR-312 (Clone 16) (Novocastra)	CC1 64'	1:50	nuclear
HER2	A0485 (Dako)	CC1 36'	1:250	membranous

TE: tris-EDTA buffer pH 9.0 at 120°C
CC1: tris-EDTA borate buffer pH 8.0-8.5 at 95°C
P1: protease 1 (highest level) at 36°C
